# Clinical Cases

**Published:** 1868-03-01

**Authors:** 


### CLINICAL CASES.

No. 1. E. M., age 25, female, presented herself for treatment, for large two-lobed fibrous tumor in the posterior claviculo-scapular region, of two years' duration. Incision was made, and it was found to be in two distinct lobes; firmly attached to the surrounding structures, clinging closely to the clavicle, and running across to spine; there was some hæmorrhage; all the arteries were preternaturally large. The entire tumor was removed, and wound dressed with Ung. Zinci. Oxid. She afterwards presented herself entirely cured.

No. 2. A. W., age 40, male. Has been a sailor, and on the coast of Africa had what is termed the "Guinea Worm." He states that a worm two inches long came out of his leg, below the knee. I believe the leg is bitten, and an egg laid therein, which is hatched within the tissue. When the worm attempts to make its exit, a small stick is passed under it, on which it is twisted, and thus drawn out. His leg is now much inflamed and swollen, and a cooling lotion was ordered to subdue the inflammation. I give this case as being rather a rare one in these parts.

No. 3. A case of exhaustion and death from hæmorrhage. J. S., age 21, male. Dec. 21st, was struck on right side of face and head with a lager beer mug, inflicting two wounds; one, two inches and a half in length, directly in temporal region, and one, one inch in length, just beneath the right eye. He bled profusely, and was carried to the hospital. The wounds were simply dressed with adhesive strips. Sixty hours after injury he left the hospital for his house, and in twelve hours afterwards was attacked with hæmorrhage from larger wound. Two hours after this I saw him and he was pulseless. His face was anemic. The bleeding was but slight, and the patient was scarcely conscious. He had evidently lost much blood, for I measured three pints, and could of course get nothing like an actual measurement of *all* he had lost. It was impossible to ligate the bleeding vessel, for it was minute and deeply laid, and had retractred greatly. Monsell's solution was freely used, and hæmorrhage arrested. Stimulants and opiates were given freely to the patient, and the following morning he was comparatively in good condition. About noon hæmorrhage again set in violently, but was arrested by means of acupressure needle and compress. This wound bled no more, but the following day the smaller wound bled excessively before it could be arrested. Erysipelas supervened on the following day, marked with violent inflammatory symptoms and raving delirium. Bleeding now occurred from his gums and nose, and three days before his death hæmatamesis set in, which was only held at abeyance with five grain doses of Subsulph. Ferri. The patent died from exhaustion from hæmorrhage occasioned by wounds. On *post-mortem* examination I found no signs of disease in any of the organs. The wounds had in no way communicated with the brain, and the orbital branch of the temporal artery was found severed. The brain was pale and bloodless; the tissues were all pale and anemic, and the thoracic and abdominal viscera were white and blanched throughout.

Yours, E. R. H.

